# Oviposition by a lycaenid butterfly onto old host parts is adaptive to avoid interference by conspecific larvae

**DOI:** 10.1371/journal.pone.0252239

**Published:** 2021-05-26

**Authors:** Yukari Mochioka, Motoaki Kinoshita, Makoto Tokuda

**Affiliations:** 1 Faculty of Agriculture, Laboratory of Systems Ecology, Saga University, Saga, Japan; 2 The United Graduate School of Agricultural Sciences, Kagoshima University, Kagoshima, Japan; University of Arkansas, UNITED STATES

## Abstract

Oviposition site selection by herbivores can depend not only on the quality of host resources, but also on the risk of predation, parasitism and interference. Females of the lycaenid butterfly *Arhopala bazalus* (Lepidoptera) lay eggs primarily on old host foliage away from fresh growth, where larval offspring live and feed. Resource availability of young host leaves seems not to affect the oviposition site preference by the females. To clarify the adaptive significance of *A*. *bazalus* oviposition behavior on old foliage, we tested three hypotheses: eggs on fresh foliage are (1) easily dropped during rapid leaf expansion (bottom-up hypothesis), (2) more likely to be attacked by egg parasitoids (top-down hypothesis), and (3) frequently displaced or injured by other herbivores (interference hypothesis). In field surveys, rates of egg dropping and parasitism by egg parasitoids were not significantly different between fresh and old host parts. However, the portions of fresh leaves on which *A*. *bazalus* eggs had been laid were cut from shoots on which conspecific larvae fed. Laboratory experiments demonstrated that eggs on young leaves were displaced in the presence of conspecific larvae and we observed that fifth instar larvae actively displaced conspecific eggs by feeding on the surrounding leaf tissue. These findings indicate that eggs laid on fresh leaves are at risk of being displaced by conspecific larvae, and support the interference hypothesis. Larval behavior is a likely evolutionary force for *A*. *bazalus* to lay eggs apart from larval feeding sites on the host plant.

## Introduction

Insect oviposition choices are important for their own fitness because they directly affect offspring survival and growth [1–3]. Females tend to lay their eggs on host plants or habitats that are the most suitable for their offspring to successfully reach the adult stage [4,5]. Positive links between oviposition site preference and offspring performance, in terms of survival, growth and development, have been detected in multiple insect species [6–12]. However, oviposition preference and larval growth and developmental performance are poorly correlated in some studies [13–15], and eggs are sometimes laid on poor-quality hosts [16,17] or on non-host plants [14,18,19].

Such lack of positive links between oviposition decisions and offspring performance could be attributed to multiple factors, including polyphagy with high larval mobility [20], temporal variations in host quality [21–24], host abundance and distribution [25,26], interaction with novel hosts [2,27] and avoidance of natural enemies [28,29]. For example, the stink bug *Acanthocoris sordidus* Thunberg (Hemiptera: Coreidae) frequently lay their eggs on non-host plants, or even on fallen leaves on the ground, requiring hatchlings to move a long distance to feeding sites on host plants [30]. In this species, egg mortality caused by parasitic wasps is lower on non-host plants than on host plants, so oviposition onto non-host plants is suggested to reduce the risk of egg parasitism [30]. As in this case, oviposition sites better for larval growth and development do not always correspond with higher offspring survival. Therefore, females might choose sites that maximize offspring success over all life stages, or their own fitness at the expense of larval performance.

**Fig 1:**
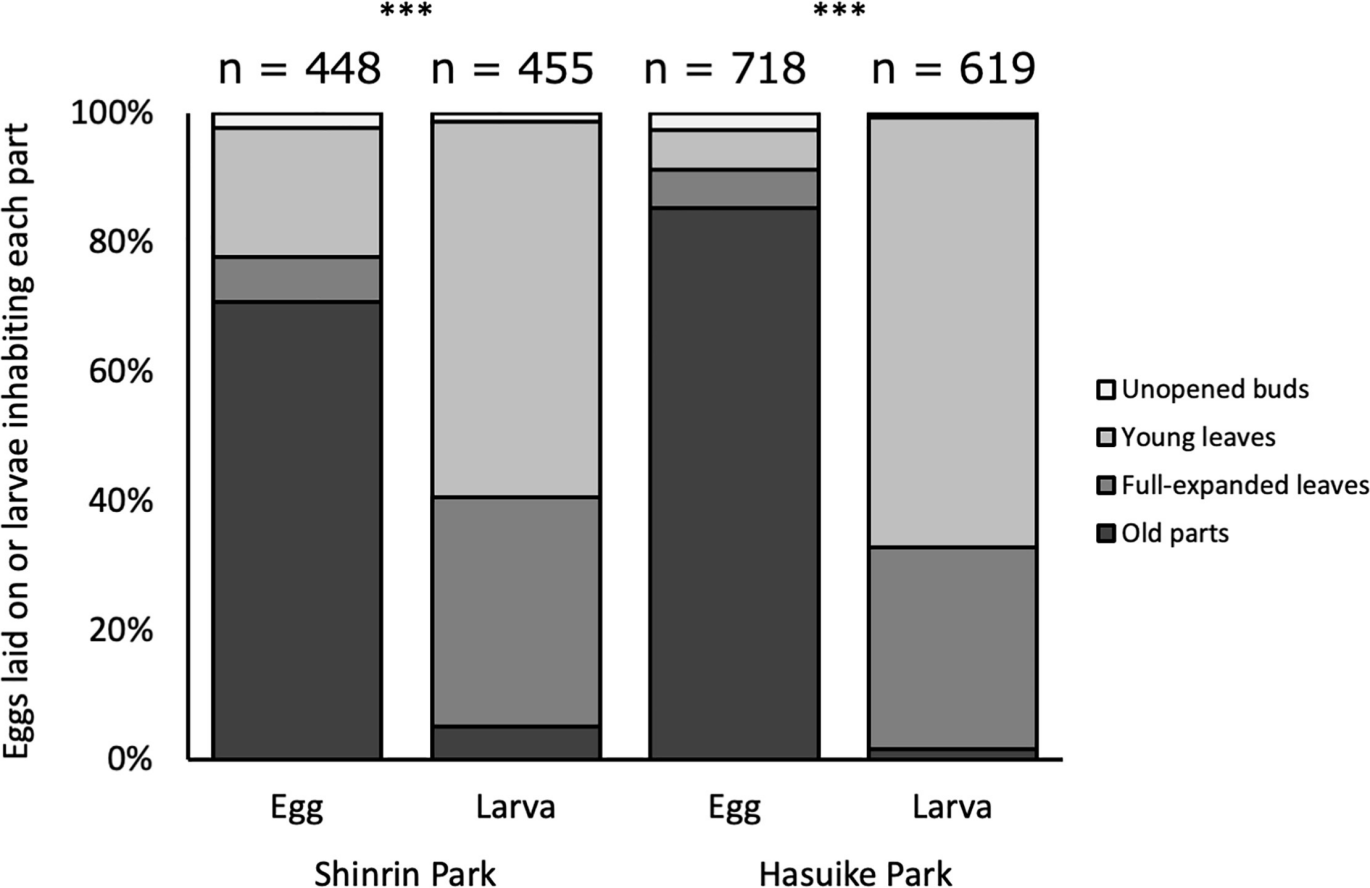
Oviposition and larval feeding sites of *A*. *bazalus* on *L*. *edulis* in Shinrin Park and Hasuike Park, Saga, Kyushu, Japan in 2015.

**Fig 2:**
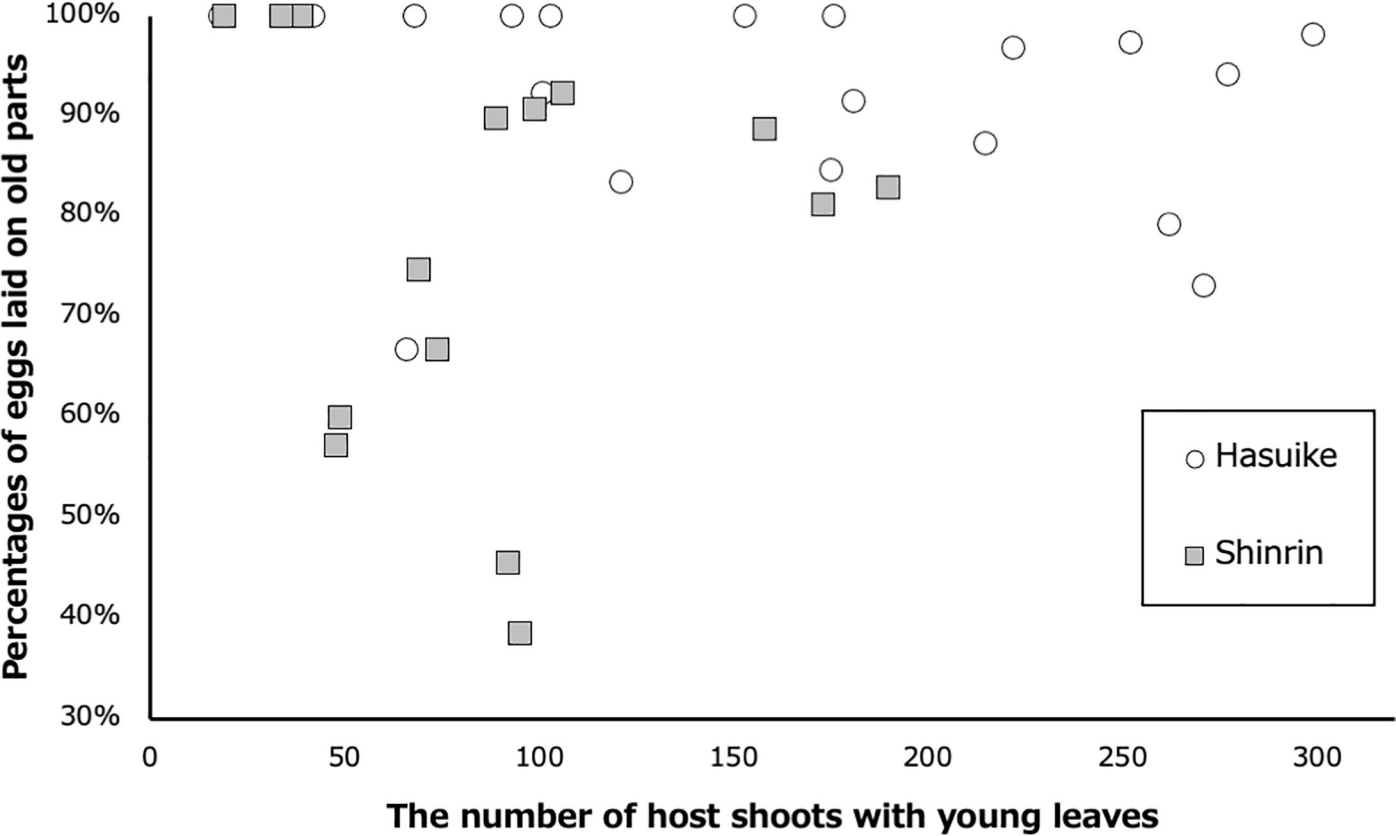
Relationship between the number of *L*. *edulis* shoots with young leaves and percentages of *A*. *bazalus* eggs laid on old host foliage in Shinrin Park and Hasuike Park, Saga, Kyushu, Japan in 2015.

Females of the lycaenid butterfly *Arhopala bazalus* (Hewitson) [= *Narathura bazalus* (Hewitson)] (Lepidoptera) lay their eggs on old leaves and stems [31], which are distant from larval feeding sites on fresh leaves [32] (see also results and Figs 1 and 2). Therefore, freshly hatched first instar larvae must move for a long distance from the oviposition site to the feeding site. This behavior implies that oviposition on old host foliage distant from larval feeding sites reduces egg mortality risk compared to oviposition onto fresh host foliage. The present study sought to determine the factor(s) dictating oviposition onto host parts distal to fresh foliage and larval feeding sites.

In the present study, we tested the following three adaptive hypotheses for oviposition site selection: Oviposition onto old host foliage is adaptive because eggs laid on fresh host foliage are (1) easily dropped during rapid leaf expansion (bottom-up hypothesis), (2) more likely to be attacked by egg parasitoids (top-down hypothesis), or (3) frequently displaced or injured by other herbivores (interference hypothesis).

To test these hypotheses, we evaluated the female oviposition preference, offspring feeding preference and offspring developmental performance of *A*. *bazalus*. We then examined dropping of *A*. *bazalus* eggs from host plant leaves in the field, compared egg parasitism rates across fresh and old host foliage, and confirmed the presence of other herbivores proximal to eggs laid on fresh foliage. Finally, we observed the behavior of conspecific larvae on fresh leaves on which eggs had been laid.

## Materials and methods

### Study sites

Field surveys were primarily conducted in Shinrin Park (N 33° 14’; E 130° 15’) and Hasuike Park (N 33° 15’; E 130° 22’), Saga Prefecture, Kyushu, Japan. Additionally, surveys were conducted in Tenjin (N 33° 16’; E 130° 18’) and on the Saga University campus (N 33° 15’; E130° 17’), Saga Prefecture, as well as in the Fudougaura (N 33° 39’; E 130° 27’), Saitozaki (N 33° 40’; E 130° 21’), and Shikanoshima (N 33° 40’; E 130° 18’) sites, Fukuoka Prefecture, Kyushu, Japan. The field studies do not involve endangered or protected species and surveys of ordinal insects are not prohibited in these sites.

### Study insect

*Arhopala bazalus* is a multivoltine species distributed in Japan, Taiwan, India (Assam and Sikkim) and Southeast Asia [33]. Although the larvae feed on several species of *Lithocarpus* (Fagaceae) trees, they are associated only with the evergreen tree *L*. *edulis* (Makino) Nakai in our study sites [33].

Typically, *A*. *bazalus* females visit host terminal buds and touch them with their antennae, then quickly fly to old leaves on the same or adjacent branches, and lay their eggs on old leaves, distal to larval feeding sites on newly emerged foliage (‘Pattern A’ in [31]). In rare cases, females alight on extended buds and directly lay eggs on the buds or fresh leaves (‘Pattern B’ in [31]). Eggs are laid not in clusters but individually. Egg, larval and pupal durations of *A*. *bazalus* are 4.0, 20.8 and 11.6 days at 25°C (15L:9D), respectively [34].

A previous study reported that 32 species of lepidopteran larvae inhabit *L*. *edulis* trees in Japan [35], but in our study sites, *A*. *bazalus* was the most common species, and at times larvae of an unidentified moth species were detected, but at relatively low densities. To the best of our knowledge, there have been no records of parasitoids associated with *A*. *bazalus* prior to our study [36].

### Categorization of host leaves

**Fig 3:**
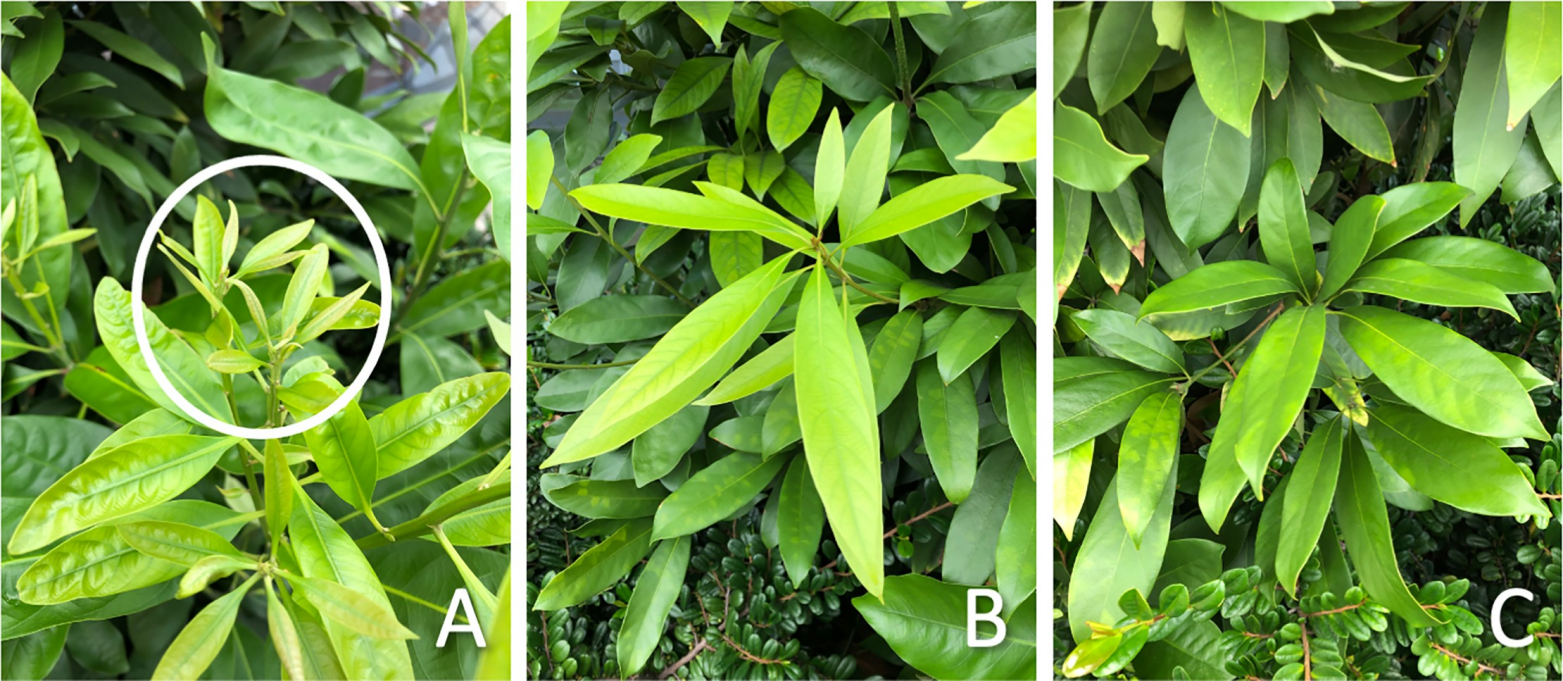
Leaf stages of *L*. *edulis*. (A) Young emerging leaves, (B) young fully expanded leaves, and (C) mature leaves.

Developmental stages of host leaves were categorized as (1) young emerging leaves (expanding), (2) young fully expanded leaves, still soft and light green, and (3) mature, dark green hardened leaves (Fig 3). Unopened buds, young emerging leaves and young fully expanded leaves were considered fresh foliage on the tree, and mature leaves and stems were considered old foliage on the tree.

### Oviposition and larval feeding sites on host trees

To examine oviposition sites and larval feeding sites of *A*. *bazalus*, 24 and 38 *L*. *edulis* trees (3–5 m tall in both sites) were investigated in Shinrin Park and Hasuike Park, respectively, every two weeks from April to November 2015. We recorded the numbers of *A*. *bazalus* eggs and larvae, and their position (fresh or old foliage) on all shoots less than 1.5 m from the ground, where the budburst frequently occurs. The numbers of unopened buds, young emerging leaves and young fully expanded leaves were also recorded across the same area of the tree as indicators of the food resources available to *A*. *bazalus* larvae. The relationship between oviposition site (fresh or old foliage) and abundance of food resources was examined to confirm whether the shortage of food resources affects oviposition sites.

### Effects of host leaf stage on larval development

We examined the effects of host leaf stage on *A*. *bazalus* larval development using eggs collected from Shinrin Park and Hasuike Park. We individually reared hatched larvae at 25°C and a 16L:8D photoperiod, and fed the larvae fresh (n = 18), or mature (n = 6) *L*. *edulis* leaves, each in their own 1.5 ml microtube. After they moulted into the fourth instar, each was transferred to a Petri dish (90 mm diameter, 15 mm height). Larval survival was confirmed daily until death or pupation.

### Effects of leaf expansion on egg dropping

Probabilities of eggs dropping at different oviposition sites were evaluated by observing unhatched eggs laid on fresh and old host shoots in the field from August to September in 2016 in Shinrin Park (4 and 10 eggs on fresh and old shoots, respectively), Hasuike Park (25 and 67 eggs on fresh and old shoots, respectively), and Fudougaura (7 and 39 eggs on fresh and old shoots, respectively). Unhatched eggs were observed daily for five successive days to confirm egg hatching or falling. The survey was conducted on shoots where other herbivores, including conspecific larvae, were absent.

### Effects of oviposition site on egg parasitism

To examine effects of oviposition site on egg parasitism, we compared parasitism rates across eggs laid on fresh and old *L*. *edulis* foliage. From July to September 2014, and from July to October 2016, unhatched eggs were collected from Shinrin Park (4 and 34 eggs on fresh and old shoots in 2014 and 6 and 37 eggs on fresh and old shoots in 2016, respectively) and Hasuike Park (10 and 13 eggs on fresh and old shoots in 2014 and 3 and 13 eggs on fresh and old shoots in 2016, respectively). We placed the eggs individually in Petri dishes (50 mm diameter, 11 mm height) and maintained the eggs at 25°C and a 14L:10D photoperiod in an incubator. We then recorded the hatching of *A*. *bazalus* larvae or the emergence of egg parasitoids from infested eggs. Eggs from which neither larvae nor parasitoids emerged were excluded from analysis.

### Effects of conspecific fifth instar larvae on egg displacement

In Shinrin Park, two *A*. *bazalus* eggs laid on fresh foliage and seven eggs laid on stems where *A*. *bazalus* fifth instar larvae were also present were marked with vinyl tape on 22 August 2015. We observed the eggs daily for five successive days to confirm egg hatching or displacement.

In the laboratory, we examined the effects of the presence of fifth instar larvae on egg displacement. *L*. *edulis* shoots on which *A*. *bazalus* eggs had been laid on fresh leaves (n = 19) or stems (n = 24) were collected from Shinrin, Fudougaura, and Hasuike Parks in September 2016. The cut end of each shoot was put in a glass vial filled with water and was placed in a plastic container (12 x 12 x 5.5 cm). Then, it was exposed to a fifth instar larva for 24 hours in an incubator at 25°C and a 14L:10D photoperiod. The fate of the eggs (fallen or retained on foliage) was then recorded.

### Statistics

We analyzed oviposition sites (fresh or old foliage), egg parasitism rates of eggs laid on fresh and old foliage and effects of fifth instar larvae on egg falling on fresh and old foliage using a Fisher’s exact probability test. The relationship between the abundance of young leaves and oviposition onto old leaves was examined by Pearson’s product-moment correlation analysis. We analyzed the effects of collection site (locality) and diet (fresh or old foliage) on the development of *A*. *bazalus* larvae using a generalized linear model (GLM) with a Poisson distribution and log-link function. We analyzed the effects of locality and oviposition site on egg falling using a GLM with a binomial distribution and logit-link function. All statistical analyses were performed using R ver. 3.5.1 [37]. Original data used in this study were shown in supporting information.

## Results

### Oviposition sites and larval feeding sites

In Shinrin Park and Hasuike Park, 70% (n = 448) and 86% (n = 718) of unhatched *A*. *bazalus* eggs were found on old foliage (mature leaves or stems), but 95% (n = 455) and 99% (n = 619) of *A*. *bazalus* larvae were found on fresh foliage (unopened buds, young emerging leaves and young fully expanded leaves) (Fig 1). The egg position (fresh vs. old foliage) was significantly different from larval site in both Shinrin Park (Fisher’s exact probability test; p < 0.001, phi = 0.676) and Hasuike Park (Fisher’s exact probability test; p < 0.001, phi = 0.836). Oviposition onto old foliage did not depend on the abundance of young leaves (Fig 2; Pearson’s product-moment correlation analysis; r^2^ = 0.001, P = 0.902 in Shinrin Park; r^2^ = 0.034, P = 0.449 in Hasuike Park).

**Fig 4:**
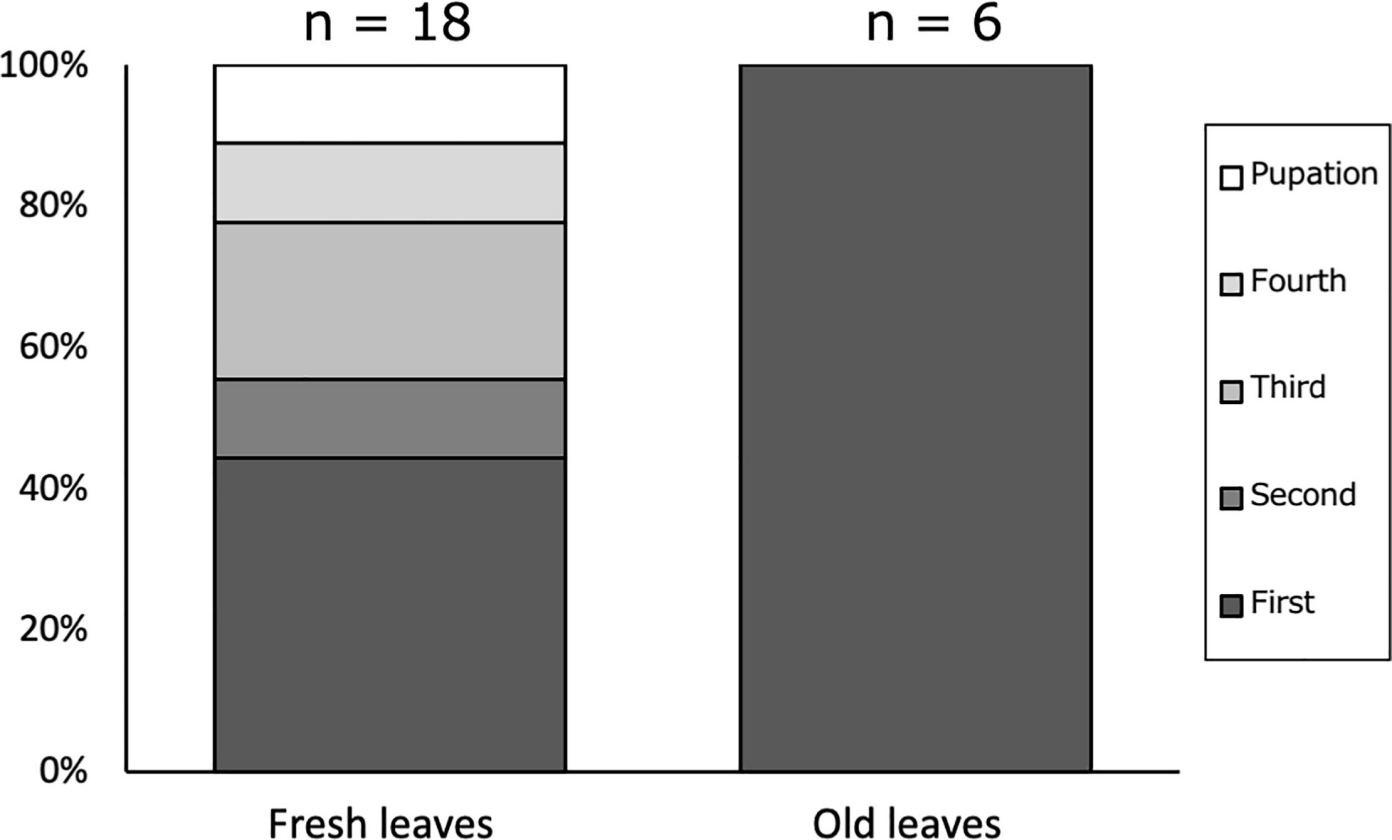
Larval survival of laboratory-reared *A*. *bazalus* fed fresh or old *L*. *edulis* leaves. First–Fourth: Larvae died at the respective instars; Pupation: Larvae successfully pupated.

In the larval rearing experiment, diet (leaf stage) significantly affected larval development, but locality and interaction between locality and diet did not affect larval development (GLM; DF = 1, χ^2^ = 5.687, p = 0.017 for diet; DF = 2, χ^2^ = 1.243, p = 0.264 for locality; DF = 2, χ^2^ = 0.171, p = 0.679 for diet × locality). All *A*. *bazalus* larvae fed mature leaves were dead at the first instar, but some of the larvae fed fresh foliage (young emerging or young fully expanded leaves) survived until pupation (Fig 4).

### Probability of egg dropping from host shoots

**Fig 5:**
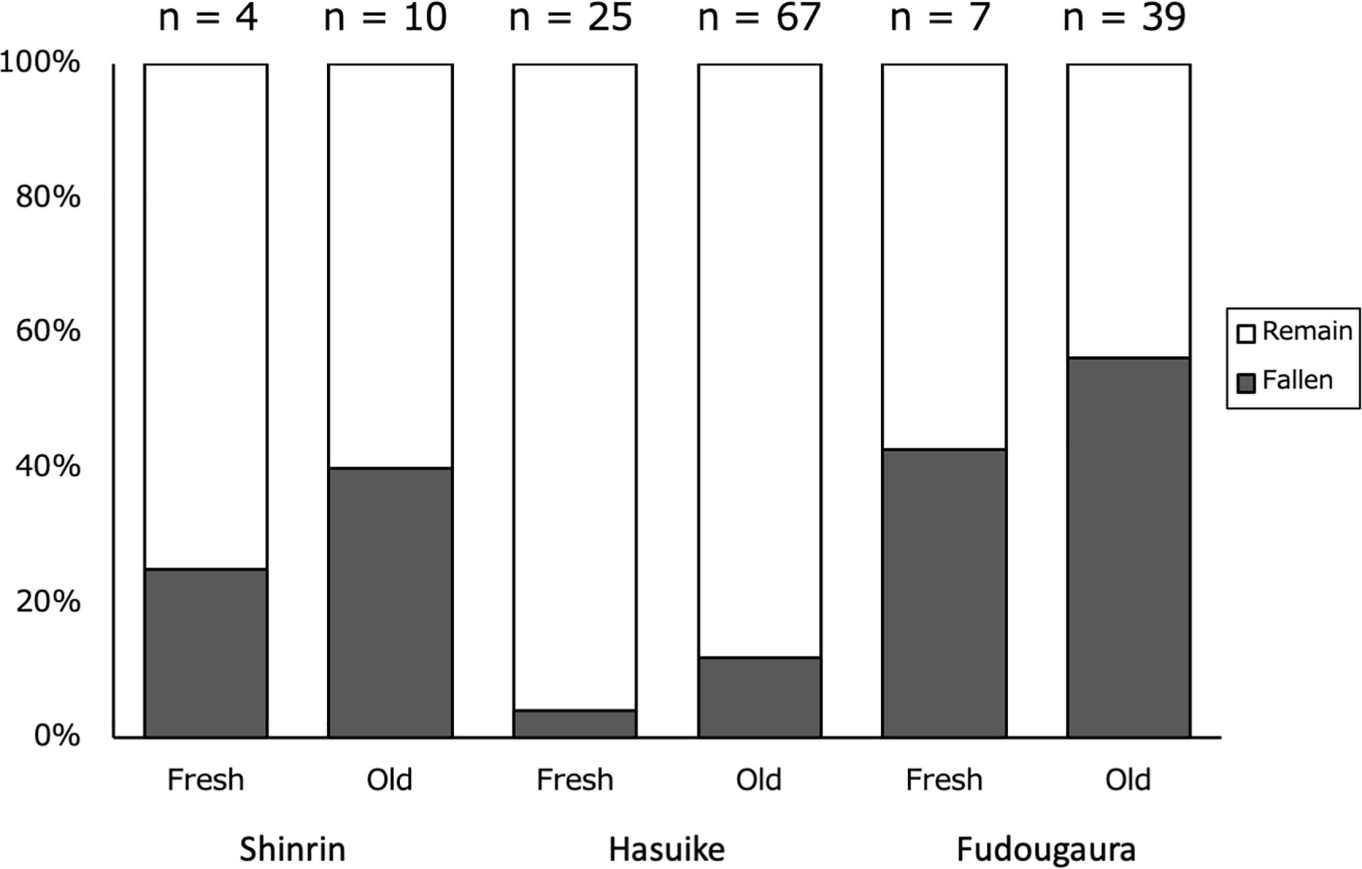
The number of eggs deposited in the field by *A*. *bazalus*, and that dropped from fresh and old *L*. *edulis* leaves in Shinrin Park and Hasuike Park (Hasuike), Saga, and Fudougaura, Fukuoka, Kyushu, Japan in 2016.

The percentages of eggs that fell within five days of oviposition are shown in Fig 5. Locality significantly affected the probability of egg dropping, but no significant differences were detected in oviposition site, or in the interaction between locality and oviposition site (GLM; DF = 2, χ^2^ = 30.77, p < 0.001 for locality; DF = 1, χ^2^ = 2.02, p = 0.155 for oviposition site; DF = 2, χ^2^ = 0.232, p = 0.891 for locality × oviposition site).

### Egg parasitoids

**Fig 6:**
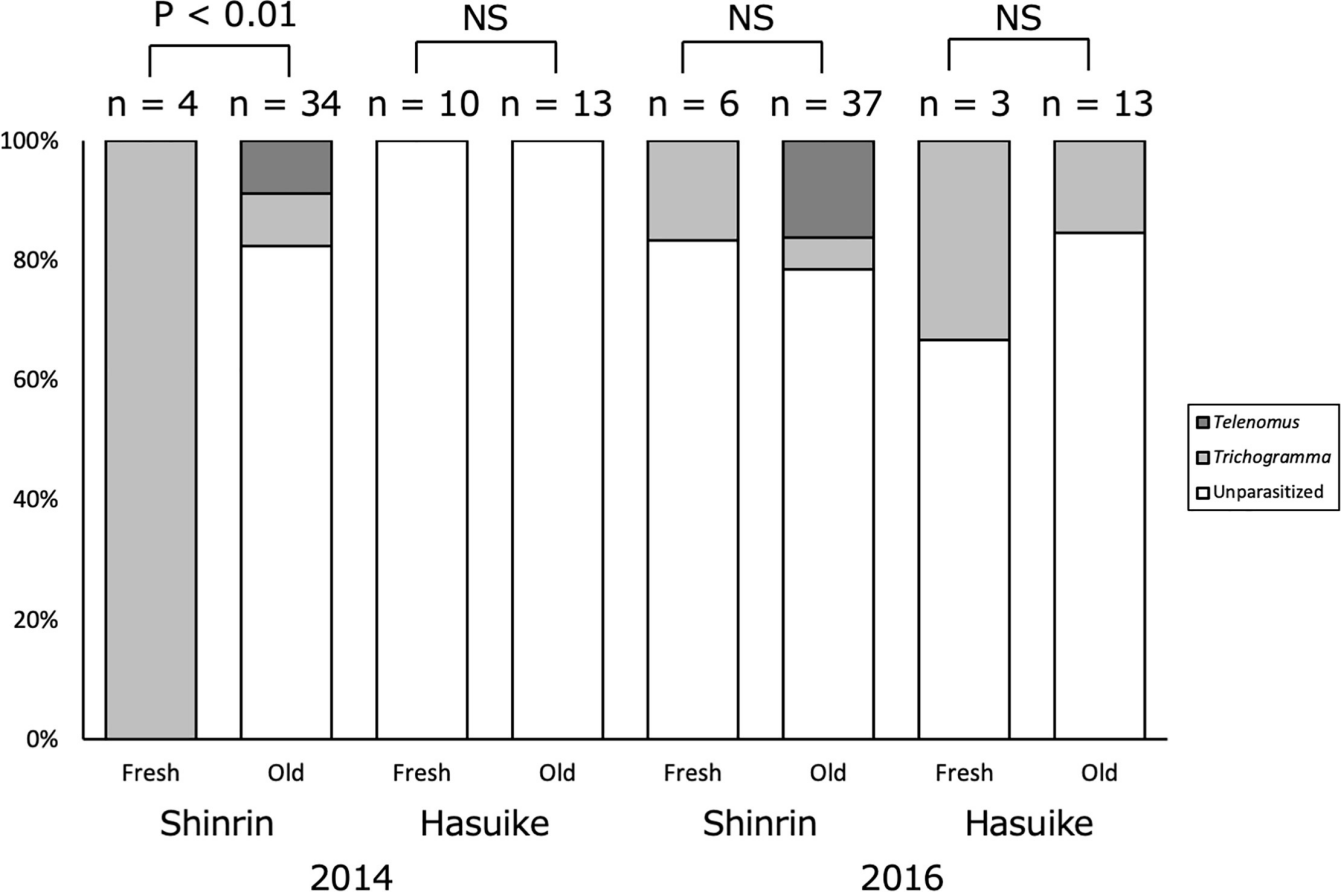
Percentages of *A*. *bazalus* eggs parasitized by *Telenomus* sp. and *Trichogramma* sp. on fresh and old *L*. *edulis* foliage collected from Shinrin Park and Hasuike Park, Saga, Kyushu, Japan in 2014 and 2016.

Two species of egg parasitoids, *Trichogramma* sp. (Hymenoptera: Trichogrammatidae) and *Telenomus* sp. (Hymenoptera: Scelionidae), emerged from *A*. *bazalus* eggs. *Trichogramma* emerged from eggs laid both on fresh and old foliage, but *Telenomus* emerged only from eggs laid on old foliage (Fig 6). In Shinrin Park, the percentages of parasitism by these parasitoids were significantly higher in eggs laid on fresh foliage than in those on old foliage in 2014 (Fisher’s exact probability test; p < 0.01; phi = 0.477), but no significant differences were detected in Shinrin Park in 2016 (p = 1.00), or in Hasuike Park in 2014 (p = 1.00) or 2016 (p = 0.489) (Fig 6).

### Effects of conspecific fifth instar larvae on egg displacement

**Fig 7:**
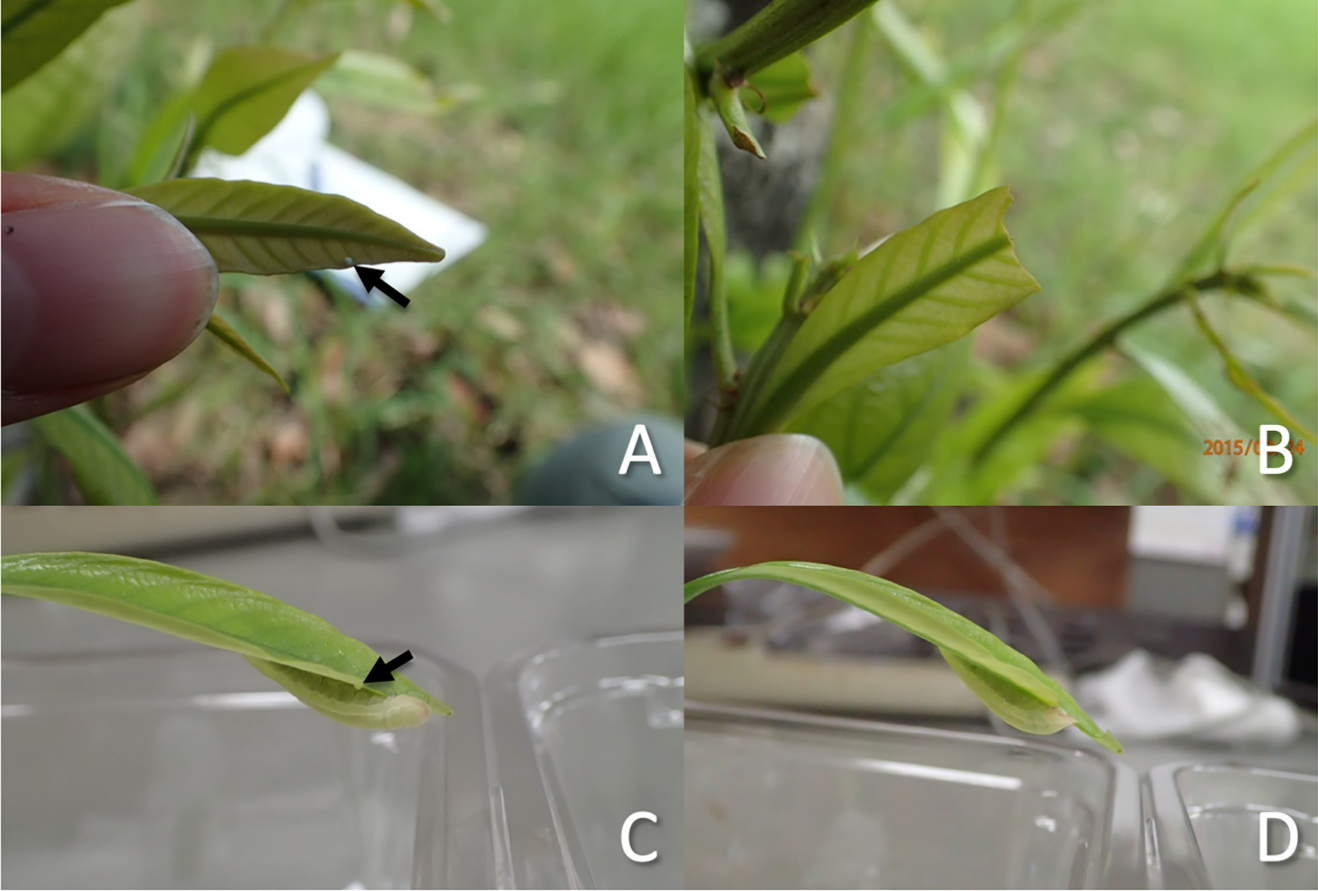
Examples of field (A-B) and laboratory (C-D) observations of *A*. *bazalus* eggs. (A) An *A*. *bazalus* egg (an arrow) laid on a young emerging *L*. *edulis* leaf, and (B) the same leaf, from which the apical part had been eaten and the egg was displaced after two days; a fifth instar *A*. *bazalus* larva (C) finding a conspecific egg (an arrow) on the host leaf and feeding on the surrounding leaf tissue and (D) displacing the egg by feeding on the surrounding leaf tissue.

In Shinrin Park, the two eggs laid on young emerging or young fully expanding leaves had been displaced and disappeared within 1–2 days due to leaf damage around the egg-laying sites by other herbivores (Fig 7A and 7B). In contrast, all seven eggs laid on stems did not disappear before hatching (Fisher’s exact probability test; p = 0.028, phi = 0.679).

In the laboratory experiment, 14 of 19 eggs on fresh leaves (73.6%) were displaced due to leaf damage of surrounding leaf tissue within 24 hours of exposure to fifth instar *A*. *bazalus* larvae, while only 3 of 24 eggs on stems (12.5%) (Fisher’s exact probability test; p < 0.001, phi = 0.574). We observed that a fifth instar larva walked around a host leaf, found the egg, and displaced it by feeding on the surrounding leaf tissue (Fig 7C and 7D).

## Discussion

In the present study, we demonstrated that most *A*. *bazalus* eggs were laid on mature leaves or stems, although *A*. *bazalus* larvae inhabited and fed only on new buds or fresh leaves. Further, oviposition site selection was not related to the abundance of young host leaves. These observations strongly suggest that oviposition onto mature leaves or stems has an adaptive advantage, which overcomes the mortality risk associated with hatchlings migrating from oviposition sites to feeding sites.

In the field, eggs laid on fresh host plant foliage seldom fell to the ground, in the absence of other herbivores, and safely hatched as did eggs laid on old foliage. This indicates that rapid host leaf expansion was not a mortality factor for *A*. *bazalus* eggs, and refutes the bottom-up hypothesis for oviposition behavior.

We found two species of hymenopteran egg parasitoids associated with *A*. *bazalus*, and showed that egg parasitism rates were not significantly lower on old foliage relative to new foliage. Indeed, *Telenomus* sp. was found only in eggs laid on old foliage in one census site. Thus, oviposition onto old foliage was not advantageous in reducing egg parasitism, refuting the top-down hypothesis.

In the field, we observed that eggs laid on fresh foliage were sometimes lost due to herbivore damage of surrounding leaf tissue when conspecific larvae were present near the eggs. In the laboratory, we demonstrated that eggs laid on young leaves were displaced in the presence of fifth instar larvae, while those laid on stems remained. Moreover, we observed in the laboratory that fifth instar larvae removed conspecific eggs by cutting off the surrounding leaf tissue. These results suggest that oviposition onto old foliage reduces the risk of egg displacement by conspecific larvae, supporting the interference hypothesis for oviposition behavior. In the present study, we observed egg-removing behavior by fifth indstar larvae, but other instar larvae could also display similar behaviors and this is a future study subject.

In Lepidoptera, *Hesperia lindseyi* (Holland) (Hesperiidae) of which larvae associated with bunchgrasses, such as *Festuca idahoensis* Elmer and *Danthonia californica* Bolander (Poaceae), growing on the ground lay eggs on arboreal lichens distant from the hosts [38]. Although the significance of this behavior has not yet intensively surveyed, it also may be adaptive to avoid mortality of eggs laid on the hosts. Larvae of some *Heliconius* (Nymphalidae) associated with the passion flowers *Passiflora* (Passifloraceae) are cannibalistic, and females seem to avoid leaves occupied by conspecifics [39]. Such behavior may facilitate the convergent evolution of egg mimic structures of some *Passiflora* plants [40,41].

As in *Heliconius* larvae, some lycaenid larvae also exhibit cannibalism. For example, *Orachrysops noibe* (Trimen) larvae feed on conspecific larvae to enhance development [42]. *Arhopala bazalus* larvae also display cannibalism, at least under laboratory rearing conditions (Y. Mochioka, unpublished observation). Therefore, the presence of larger conspecific larvae on the same leaf could increase the mortality risk for smaller larvae. In our study, however, eggs laid on stems safely hatched even when exposed to conspecific larvae. In *Anthocharis scolymus* (L.) (Lepidoptera: Pieridae), eggs suffer increased mortality due to egg cannibalism by conspecific larvae [43], and older females tend to avoid oviposition onto egg-loaded host plants [44]. In our case, fifth instar *A*. *bazalus* larvae did not directly injure the eggs, but instead cut off surrounding leaf tissue to displace them. Such differences in larval behavior are interesting and further studies are needed to clarify which factors are related to the evolution of different behaviors.

*A*. *bazalus* females first confirm the presence of fresh shoots on host trees, and then oviposit onto old host foliage [31]. Even though eggs laid on new stems are seldom displaced, females tended to avoid oviposition on these sites regardless of the presence of conspecific larvae, but instead preferred oviposition on older host foliage. This could be due in part to trade-offs between spending time to find better sites and laying more eggs, as has been shown in several other butterflies [45–48]. *A*. *bazalus* egg size (0.65 mm diameter, 0.40 mm height) is smaller than that of a similar-sized and sympatrically distributed congener, *Arhopala japonica* (Murray) (0.80 mm diameter, 0.43 mm height) [49]. This suggests that *A*. *bazalus* has a strategy to lay as many eggs as possible rather than to choose better sites for fewer offspring. On the other hand, smaller eggs and hatchlings could result in lower starvation tolerance. The differences in oviposition sites, starvation tolerance, and hatchling mobility between *A*. *bazalus* and *N*. *japonica* in relation to adult longevity, fecundity, host resources, natural enemies and larval competition will be the topic of future investigations.

In conclusion, we demonstrated that the oviposition by *A*. *bazalus* females onto old host parts distant from larval feeding site has an adaptive advantage to avoid egg mortality due to interference by conspecific larvae.

## Supporting information

S1 File (XLSX)

## References

[1] Jaenike J. On optimal oviposition behavior in phytophagous insects. Theor Popul Biol. 1978; 14: 350–356. 10.1016/0040-5809(78)90012-6 751265

[2] Thompson JN. Evolutionary ecology of the relationship between oviposition preference and performance of offspring in phytophagous insects. Entomol Exp Appl. 1988; 47: 3–14.

[3] Mayhew PJ. Herbivore host choice and optimal bad motherhood. Trends Ecol Evol. 2001; 16: 165–167. 10.1016/s0169-5347(00)02099-1 11245932

[4] Videla M, Valladares GR, Salvo A. Choosing between good and better: optimal oviposition drives host plant selection when parents and offspring agree on best resources. Oecologia. 2012; 169, 743–751. 10.1007/s00442-011-2231-6 22246471

[5] Jones LC, Rafter MA, Walter GH. Insects allocate eggs adaptively across their native host plants. Arthropod-Plant Interact. 2019; 13: 181–191.

[6] Craig TP, Itami JK, Price PW. A strong relationship between oviposition preference and larval performance in a shoot-galling sawfly. Ecology. 1989; 70: 1691–1699.

[7] Barker AM, Maczka CJM. The relationships between host selection and subsequent larval performance in three free-living graminivorous sawflies. Ecol Entomol. 1996; 21: 317–327.

[8] Craig TP, Ohgushi T. Preference and performance are correlated in the spittlebug *Aphrophora pectoralis* on four species of willow. Ecol Entomol. 2002; 27: 529–540.

[9] Forister ML. Oviposition preference and larval performance within a diverging lineage of lycaenid butterflies. Ecol Entomol. 2004; 29: 264–272.

[10] Scheirs J, Zoebisch TG, Schuster DJ, De Bruyn L. Optimal foraging shapes host preference of a polyphagous leafminer. Ecol Entomol. 2004; 29: 375–379.

[11] Gripenberg S, Mayhew PJ, Parnell M, Roslin T. A meta-analysis of preference-performance relationships in phytophagous insects. Ecol Lett. 2010; 13: 383–393. 10.1111/j.1461-0248.2009.01433.x 20100245

[12] Sidhu JK, Stout MJ, Blouin DC. Performance and preference of sugarcane borer, *Diatraea saccharalis*, on rice cultivars. Entomol Exp Appl. 2013; 149: 67–76. 10.1017/S0007485313000369 23830057

[13] Janz N. Evolutionary ecology of oviposition strategies. In: Hilker H, Meiners T, editors. Chemoecology of insect eggs and egg deposition. Berlin: Blackwell; 2002. pp. 349–376.

[14] Verdon A, Margraf N, Davison AC, Rahier M, Naisbit RE. Conserved oviposition preferences in alpine leaf beetle populations despite host shifts and isolation. Ecol Entomol. 2007; 32: 62–69.

[15] Adar S, Dor R. Mother doesn’t always know best: maternal wormlion choice of oviposition habitat does not match larval habitat choice. Behav. Processes. 2018; 147: 1–8. 10.1016/j.beproc.2017.12.002 29221673

[16] Singer MS, Stireman JO III. Does anti-parasitoid defense explain host-plant selection by a polyphagous caterpillar? Oikos. 2003; 100: 554–562.

[17] Moon DC, Stirling P. Trade-off in oviposition strategy: choosing poor quality host plants reduces mortality from natural enemies for a salt marsh planthopper. Ecol Entomol. 2006; 31: 236–241.

[18] Singer MC. Butterfly—hostplant relationships: host quality, adult choice and larval success. In Vane-Wright R, Ackery PR, editors. The biology of butterflies. London: The Royal Entomological Society, Academic Press; 1984. pp. 81–88.

[19] Wiklund C. Egg-laying patterns in butterflies in relation to their phenology and the visual apparency and abundance of their host plants. Oecologia. 1984; 63: 23–29. 10.1007/BF00379780 28311161

[20] Kumashiro S, Matsukura K, Adachi S, Matsumura M, Tokuda M. Oviposition site preference and developmental performance of a gall-inducing leafhopper on galled and non-galled host plants. Entomol Exp Appl. 2016; 160: 18–27.

[21] Rausher MD. Larval habit at suitability and ovipositi on preference in three related butterflies. Ecology. 1979; 60: 503–511.

[22] Gripenberg S, Salminen JP, Roslin T. A tree in the eyes of a moth—temporal variation in oak leaf quality and leafminer performance. Oikos. 2007; 116: 592–600.

[23] Roslin T, Salminen JP. (2009) A tree in the jaws of a moth—temporal variation in oak leaf quality and leaf-chewer performance. Oikos. 2009; 118: 1212–1218.

[24] Ruhnke H, Schädler M, Klotz S, Matthies D, Brandl R. (2009) Variability in leaf traits, insect herbivory and herbivore performance within and among individuals of four broad-leaved tree species. Basic Appl Ecol. 2009; 10: 726–736.

[25] Williams KS, Lincoln DE, Ehrlich PR. The coevolution of *Euphydryas chalcedona* butterflies and their larval host plants. Oecologia. 1983; 56: 323–329. 10.1007/BF00379707 28310211

[26] Rowell-Rahier M. The food plant preferences of *Phratora vitellinae* (Coleoptera: Chrysomelidae) A. Field observations. Oecologia. 1984; 64: 369–374. 10.1007/BF00379135 28311453

[27] Chew FS. Coevolution of pierid butterflies and their cruciferous foodplants. II. The distribution of eggs on potential foodplants. Evolution. 1977; 31: 568–579. 10.1111/j.1558-5646.1977.tb01045.x 28563490

[28] Singer M. S, Rodrigues D. Stireman JOIII, Carrière Y. Roles of food quality and enemy-free space in host use by a generalist insect herbivore. Ecology. 2004; 85: 2747–2753.

[29] Nakajima Y, Fujisaki K. Fitness trade-offs associated with oviposition strategy in the winter cherry bug, *Acanthocoris sordidus*. Entomol Exp Appl. 2010; 137: 280–289.

[30] Nakajima Y, Nakagawa R, Fujisaki K. Interactions between the winter cherry bug *Acanthocoris sordidus* (Hemiptera: Coreidae) and its egg parasitic wasps. Appl Entomol Zool. 2012; 47: 35–44.

[31] Sunose T, Nakagawa K. Egg-laying behavior of a lycaenid butterfly, *Narathura bazalus turbata* Butler (Lepidoptera, Lycaenidae) and the spatial distribution of the eggs. Kontyû. 1984; 52: 229–237.

[32] Fukuda H. Insects’ life in japan Vol. 3 Butterflies. Ohsaka: Hoikusha Publishers Co. Ltd; 1972. (In Japanese).

[33] Shirôzu T. The Butterflies of Japan in Color. Tokyo: Gakken; 2006 (In Japanese).

[34] Asou H, Inoue T, Koyama T. Effect of thermoperiod on immature development of powdered oakblue, *Narathura bazalus* (Hewitson) (Lepidoptera: Lycaenidae). Jpn J Appl Entomol Zool. 2006; 50: 241–246. (In Japanese with English summary).

[35] Teramoto N. Catalogue of host plants of lepidopterous insects in Japan (Fagaceae). Bull. Shiga Agric. Exp. Sta. Ext. Issue. 1993; 1: 1–185.

[36] Noyes JS. Universal Chalcidoidea Database. World Wide Web electric publication. 2019 [cited 2019 December 15]. Available from: http://www.nhm.ac.uk/chalcidoids.

[37] R Core Team. R: A language and environment for statistical computing. R Foundation for Statistical Computing, Vienna; Austria. 2018. Available from: http://www.R-project.org/.

[38] MacNeill CD. The skippers of the genus *Hesperia* in western North America (Lepidoptera: Hesperiidae). Univ. Calif. Publ. Entomol. 1964; 35: 1–230.

[39] de Castro ECP, Zagrobelny M, Cardoso MZ, Bak S. The arms race between heliconiine butterflies and *Passiflora* plants–new insights on an ancient subject. Biol. Rev. 2018; 98: 555–573. 10.1111/brv.12357 28901723

[40] Gilbert E. The coevolution of a butterfly and a vine. Sci. Amer. 1982; 247: 110–121. 10.1038/scientificamerican0782-110 7134958

[41] Vanderplank J, Boender R. *Passiflora poslae*. Curtis’s Bot. Mag. 2008; 25: 237–244.

[42] Edge D, Hamburg H. Larval feeding behaviour and myrmecophily of the Brenton blue, *Orachrysops niobe* (Trimen) (Lepidoptera: Lycaenidae). J Res Lepidoptera. 2010. 42: 31–33.

[43] Kinoshita M. Effects of time-dependent intraspecific competition on offspring survival in the butterfly, *Anthocharis scolymus* (L.) (Lepidoptera: Pieridae). Oecologia. 1998; 114: 31–36. 10.1007/s004420050416 28307554

[44] Nomakuchi S. Masumoto T, Sawada K, Sunahara T, Itakura N, Suzuki N. Possible age-dependent variation in egg-loaded host selectivity of the pierid butterfly, *Anthocharis scolymus* (Lepidoptera: Pieridae): A field observation. J Ins Behav. 2001; 14: 451–458.

[45] Wiklund C, Persson A. Fecundity, and the relation of egg weight variation to offspring fitness in the speckled wood butterfly *Pararge aegeria*, or why don’t butterfly females lay more eggs? Oikos. 1983; 40: 53–63.

[46] Doak P, Kareiva P, Kingsolver J. Fitness consequences of choosy oviposition for a time-limited butterfly. Ecology. 2006; 87: 395–408. 10.1890/05-0647 16637365

[47] Jaumann S, Snell-Rood EC. Trade-offs between fecundity and choosiness in ovipositing butterflies. Anim Behav. 2017; 123: 433–440.

[48] Chittka L, Skorupski P, Raine NE. Speed–accuracy tradeoffs in animal decision making. Trends Ecol Evol. 2009; 24: 400–407. 10.1016/j.tree.2009.02.010 19409649

[49] Teshirogi M. An Illustrated Book of the Japanese Lycaenidae. Tokyo: Tokai University Press; 1997. (In Japanese).

